# Post-COVID-19 Guillain-Barré Syndrome: A Case Report With Literature Review

**DOI:** 10.7759/cureus.21246

**Published:** 2022-01-14

**Authors:** Nidhi Kaeley, Ankita Kabi, Aadya Pillai, Takshak Shankar, Salva Ameena M S

**Affiliations:** 1 Emergency Medicine, All India Institute of Medical Sciences, Rishikesh, IND; 2 Emergency Medicine (Anaesthesiology), All India Institute of Medical Sciences, Rishikesh, IND

**Keywords:** guillain-barré syndrome, neurological disease, peripheral neuropathy, gbs, sars-cov-2 infection, covid-19

## Abstract

Coronavirus disease 2019 (COVID-19) predominantly affects the respiratory system with manifestations ranging from a mild upper respiratory tract infection to severe acute respiratory distress syndrome. Neurological manifestations of COVID-19 are mainly thrombotic manifestations affecting the nervous system; however, demyelinating manifestation has been less defined. Although some recent studies have described the association between COVID-19 and Guillain-Barré syndrome (GBS), the strength of association and features of GBS in this setting are not yet clear. Here, we report one adult case of COVID-19 infection presenting with acute GBS, which was not preceded by any other respiratory, gastrointestinal, or other systemic infections. We performed a literature search in Medline via PubMed using the keywords or MeSH terms “COVID-19” or “SARS-CoV-2” and “Guillain-Barré syndrome” and “AIDP” and “AMAN,” “Miller-Fischer syndrome” or “MFS.” We reviewed 99 case reports, 38 reviews, and two meta-analyses. Several published reports have described a possible association between GBS and COVID-19 infection.

## Introduction

Severe acute respiratory syndrome coronavirus 2 (SARS-CoV-2), first discovered in Wuhan, China, evolved into a pandemic that has drastically affected the lives of millions of people. Coronavirus disease 2019 (COVID-19) caused by the virus predominantly affects the respiratory system with manifestations ranging from a mild upper respiratory tract infection to severe acute respiratory distress syndrome and septic shock, along with various neurological manifestations. While there have been several reports of thrombotic manifestations affecting the nervous system, a demyelinating manifestation has been uncommonly reported.

Guillain-Barré syndrome (GBS) is an acute-onset inflammatory disorder of the peripheral nervous system [1]. An infection often precedes the disease by 14 days [2]. Neurological manifestations include ascending muscle paralysis, sensory disturbances, and autonomic dysfunction. Various causative organisms have been reported previously in patients with GBS including *Campylobacter jejuni*, cytomegalovirus, *Mycoplasma pneumoniae*, Epstein-Barr virus, and influenza virus [3]. SARS-CoV-2 infection as a preceding illness to GBS has also been added to the list. Here, we present a case of GBS in a patient with a history of COVID-19 infection who presented to our emergency department and compare the findings with similar reports in the literature. The objective of the literature review was to examine the strength of association between COVID-19 and GBS.

## Case presentation

A 40-year-old female with no known comorbidities, without any medication history, presented to the emergency department with complaints of weakness of bilateral upper and lower limbs for 14 days associated with loss of bladder and bowel sensation for four days. She provided a history of fever one month back. Fever was mild to moderate, intermittent, not associated with chills, rigors, and evening rise of temperature. She also had a dry cough, myalgia, headache, and anosmia. She did not complain of shortness of breath. She was started on paracetamol 650 mg orally three times daily for two days and azithromycin 500 mg orally once daily for five days. She was diagnosed with a category I COVID-19 infection using the real-time reverse transcription-polymerase chain reaction technique. Category I infection was as per the All India Institute of Medical Sciences (AIIMS)/Indian Council of Medical Research (ICMR) National Task Force classification. However, her oxygen saturation remained above 94%. Her fever subsided after 10 days. On day 12 after the subsidence of the fever, she developed numbness and paresthesia of bilateral feet, which ascended to bilateral palms over one day. Two days later, she developed weakness in bilateral upper and lower limbs and could only walk with support. She had difficulty standing from a squatting position and difficulty in buttoning and unbuttoning, suggesting both proximal and distal weakness. The weakness of bilateral upper limbs occurred one day after the weakness of bilateral lower limbs. On day 15 after the subsidence of the fever, she complained of sudden-onset loss of bowel and bladder sensation leading to urinary retention, for which she consulted a local practitioner. She was then referred to our hospital for further management on day 17. On presentation, her bowel and bladder incontinence had begun to improve but the weakness was static. She had no history of diabetes, hypertension, or smoking. There was no history of trauma, ptosis, diplopia, dysarthria or dysphagia, or food poisoning.

On examination, she was afebrile and conscious of time, place, and person. Her pulse rate was 85 beats per minute, blood pressure was 120/80 mmHg, respiratory rate was 18 breaths per minute, and oxygen saturation was 96% on room air. Central nervous system examination was suggestive of quadriparesis. Power of bilateral lower limb was 3/5 at hip and thigh muscles and 4/5 at the ankle. Power of bilateral upper limb was 4+/5 on shoulders and arms and 5/5 on the handgrip. Deep tendon reflexes were absent. Bilateral plantar reflex was flexor. Paresthesia was present in bilateral distal one-third of lower limbs. Superficial sensory functions including pain, touch, temperature, and deep sensory functions, including joint sense, position sense, and vibration sense, were intact. Cranial nerve examination was normal. She had no sensory spinal level and no signs of meningeal irritation. Cardiovascular, abdominal, and respiratory system examinations did not reveal any abnormality.

The hematological and biochemical parameters were within the normal range. Cerebrospinal fluid (CSF) examination showed neutrophilic leukocytosis and elevated protein level (protein: 219 mg/dL, sugar: 58 mg/dL, total leukocyte count: 60/mm^3^, differential leukocyte count: N63L37) suggested albuminocytological dissociation. Magnetic resonance imaging of the whole spine revealed spondylodegenerative changes in the cervical and lumbar spine without any nerve root compression. Nerve conduction velocity study revealed prolonged distal latency with reduced amplitude of compound muscle action potentials in the bilateral median, ulnar, peroneal, and tibial nerves, as well as prolonged F-latency in bilateral upper limbs. This suggested demyelinating sensorimotor polyneuropathy affecting all four limbs (lower limbs > upper limbs). Other investigations were within normal limits.

Based on the above-mentioned findings, a diagnosis of GBS-acute inflammatory demyelinating polyneuropathy was made. Because the patient presented after around two weeks of the onset of symptoms, which showed signs of improvement, the decision to administer intravenous immunoglobulin (IVIG) was deferred. She was managed conservatively and recovered fully by day four of admission. She was discharged on day five in a stable condition and had normal vitals on follow-up.

## Discussion

COVID-19 infection is caused by SARS-CoV-2, a positive-sense, single-stranded RNA beta coronavirus. The spike protein of the virus mainly aids in transmissibility. The virus targets angiotensin-converting enzyme-2 (ACE-2) receptors of the respiratory epithelial cells. Multiple neurological manifestations have been reportedly associated with SARS-CoV-2 infection, such as multiple sclerosis, cerebrovascular disease, acute encephalitis, acute disseminated encephalomyelitis, and polyneuropathy. GBS has been reported in less than 0.5% of SARS-CoV-2 infections [4]. However, an accurate estimation of the incidence of GBS in COVID-19 patients is unknown because the potential association remains uncertain.

The underlying pathogenesis of COVID-19 causing GBS is under investigation. GBS is an inflammatory disorder caused by an altered host response to infections leading to molecular mimicry and resultant damage to peripheral nerves and nerve roots. The dysregulated host response leads to the activation of the complement system, infiltration of macrophages, and edema of peripheral nerve roots and cell bodies. SARS-CoV-2 infection provokes cytokine immune response by activating Th1 cells and CD14+ and CD 16+ monocytes. This leads to the production of increased quantities of interleukin (IL)-6, tumor necrosis factor-alpha (TNF-α), and other miscellaneous cytokines.

The SARS-CoV-2 infection also leads to the activation of nuclear factor-kappa B with the help of pattern recognition proteins. The virus binds to the ACE-2 receptor on the host cell, which results in decreased ACE-2 and increased angiotensin II expression. Activation of angiotensin type II-angiotensin type I receptor further stimulates TNF-α and soluble IL-6 rheumatoid arthritis form. Thus, various cytokines are involved such as vascular endothelial growth factor, monocyte chemoattractant protein-1, IL-8, and IL-6. The latter results in the impairment of innate and acquired immune responses [5,6].

GBS mainly affects the peripheral nervous system with an approximate global incidence of 1-2 per 100,000 person-years. It is an acute-onset ascending flaccid areflexic paralysis. Diagnosis is usually made on the basis of clinical history, examination, CSF analysis, and electrophysiological studies. GBS presents as progressive relatively symmetrical weakness with decreased or absent myotatic reflexes, and symptoms reach maximal intensity within four weeks of onset. Other possible causes must be excluded. The findings on CSF analysis show albuminocytologic dissociation, which is an elevation in CSF protein without an elevation in white blood cells. Protein levels may be normal early but are elevated by the end of the second week of symptoms in 90% of cases. According to nerve conduction studies and presentations, the described variants of GBS include acute inflammatory demyelinating polyneuropathy (AIDP), acute motor axonal neuropathy (AMAN), acute motor-sensory axonal neuropathy (AMSAN), and Miller Fisher syndrome (MFS) [7,8].

Our patient presented with acute-onset flaccid areflexic motor sensory quadriparesis with a history of COVID-19 infection approximately two weeks prior to symptom onset. A nerve conduction study confirmed our diagnosis, which revealed an AIDP variant of GBS.

We performed a literature search in Medline via PubMed using the following keywords or MeSH terms: “COVID-19” or “SARS-CoV-2” and “Guillain-Barré syndrome” and “AIDP” and “AMAN,” “Miller-Fischer syndrome” or “MFS.” We found a substantial number of reported cases and case series of COVID-19 infections presenting with GBS. In total, we reviewed 99 case reports, 38 reviews, and two meta-analyses. The important studies are listed in Table 1.

**Table 1:** Study details and results of articles included in the literature review COVID-19: coronavirus disease 2019; GBS: Guillain-Barré syndrome; SARS-CoV-2: severe acute respiratory syndrome coronavirus 2; AMAN: acute motor axonal neuropathy; AMSAN: acute motor and sensory axonal neuropathy; CIM: critical illness myopathy; CPK: creatine phosphokinase; ICU: intensive care unit; CSF: cerebrospinal fluid; MFS: Miller Fisher syndrome; AIDP: acute inflammatory demyelinating polyradiculopathy

Author	Setting	Results
Godoy-Santín et al. [9]	96 patients with COVID-19 admitted with neurological complications	Only one patient had GBS. Other diagnoses included delirium, stroke in 24, critical illness, polyneuropathy, myopathy, seizures, brachial plexopathy, compressive neuropathies, encephalitis, and vasculitis
López-Hernández et al. [10]	A comparative analysis between SARS-CoV-2-related GBS and non-SARS-CoV-2 patients and a comparison with 2019 cases	When comparing patients with GBS in 2020 versus patients in 2019, the study observed a decrease in the previous infection history during 2020 and a decrease in previous respiratory infection, as well as a higher frequency of cranial nerve involvement and albuminocytologic dissociation
Eslamian et al. [11]	Six patients with COVID-19 and concomitant quadriparesis	Three axonal variants of GBS, including two cases of AMAN, one case of AMSAN, three cases of myopathies, including one combination of critical illness neuropathy/critical illness myopathy, one CIM, and one acute polymyositis
Islam et al. [12]	A 40-year-old patient with features of severe SARS-CoV-2 pneumonia and high serum CPK	After 10 days of mechanical ventilation, unsuccessful weaning was evaluated, and the patient was diagnosed to have skeletal myositis
Travi et al. [13]	Retrospective study of neurological manifestations in COVID-19 involving 901 patients	Of the patients, mental confusion/dizziness in 6.8%, stroke 5.9%, dysgeusia/anosmia 9.1%, seizure 2.1%, syncope 9%, headache 4.3%, encephalitis 0.6%, psychomotor agitation 2.9%, and post-infective encephalitis/neuropathy 0.8%. According to the severity of COVID-19, the presence of any neurologic involvement was higher among those with a moderate disease compared to those with severe or critical disease
Berra et al. [14]	Post-COVID-19 neuropathies in 10 patients	The study hypothesized that their pathogenesis is indirectly related to COVID-19 and predominantly due to prolonged maintenance of abnormal postures, ICU treatment, thrombotic complications in coagulopathy, endotheliopathy, and/or vasculitis involving vasa nervorum
Sedaghat et al. [15]	A case of GBS two weeks after COVID-19 infection	The first reported case of GBS post-COVID-19 infection
Keddie et al. [16]	Epidemiological and cohort study to investigate any causative association between COVID-19 infection and GBS	There were no significant differences in the pattern of weakness, time to nadir, neurophysiology, CSF findings, or outcome between the groups. The study found no epidemiological or phenotypic clues of SARS-CoV-2 being causative of GBS
Ottaviani et al. [17]	A case with rapidly progressive flaccid paralysis with unilateral facial neuropathy after a few days of mild respiratory symptoms	The evolution of the clinical picture does not support the typical post-infectious pattern of GBS and instead resemble a form of acute para-infectious paralysis
Zito et al. [18]	A case of post-COVID-19 GBS	AMSAN variant of GBS was seen in the patient
McDonnell et al [19]	A patient with a recurrent case of GBS occurring secondary to COVID-19 infection	GBS severity was enhanced compared to prior episodes
Nanda et al. [20]	A case series of four patients	Only one patient had cranial nerve involvement and two developed weakness after 10 days of fever onset. Two were axonal variants and others were demyelinating variant
Assini et al. [21]	A case report of two patients	One had the MFS variant and another had AMSAN
Fernández-Domínguez et al. [22]	A case report	MFS variant was found in the patient
El Otmani et al. [23]	A case report	AMSAN variant was described
Toscano et al. [24]	A case series of five patients	Two cases of AIDP, one of AMAN, two of AMSAN
Lascano et al. [25]	A case series of three patients	All patients had AIDP
Mozhdehipanah et al. [26]	A case series of three patients	Two cases of AIDP and one of AMSAN were found
Caress et al. [27]	A review of 37 published cases of GBS associated with COVID-19	Clinical presentation and severity of cases are similar to classic GBS. The electrodiagnostic study showed a demyelinating pattern in half the cases of GBS with COVID-19 infection
Abu-Rumeileh et al. [28]	A review on 73 cases of GBS	The study noted the same findings of classic GBS in COVID-19 patients. Overall, 71% of the patients showed albuminocytological dissociation. A majority had involvement of both the motor and sensory systems. The underlying mechanism of cytokine release as part of the inflammatory storm brings up the possibility of identification of a new biomarker that helps in predicting or even early detection of GBS
Filosto et al. [29]	An observational study on the incidence of GBS during COVID-19 outbreak in northern Italy	The study showed an increased incidence of GBS during the COVID-19 outbreak in northern Italy. The study also noticed that GBS following COVID-19 infection was more severe

There was co-existence of GBS and COVID-19 infection in many previous studies [24,30-32]. Although both genders were affected, a male predominance (68.9%) was observed, reflecting the gender epidemiology of COVID-19. The median patient age was 57 (49-70) years [33].

In patients with GBS, the rapid development of diaphragmatic weakness can result in reduced lung compliance and increased intrapulmonary shunting. These changes can cause rapid worsening of hypoxemia in sick patients. Delayed diagnosis of GBS is associated with higher neurological disability and high mortality; hence, early detection and prompt therapy can prevent such adverse effects [34]. Underdiagnosis is expected in mechanically ventilated patients; thus, an early diagnosis of diaphragmatic weakness should be aimed for.

An observational study by Filosto et al. showed an increased incidence of GBS during a COVID-19 outbreak in northern Italy; they also noticed that GBS following COVID-19 infection was more severe [29]. A study by Fragiel et al. in Spain also reported increased frequency and severity of GBS in COVID-19 patients [35]. The increased severity urges us to include detailed neurological evaluation in COVID patients, thus helping early diagnosis and prompt treatment. This should improve the overall outcome of the patient.

The treatment options include either IVIG or plasma exchange. The exact mechanism of action in the treatment of GBS has not been proven. IVIG is thought to act via its immune-modulating action. IVIG is administered at a dose of 2 g/kg over five days [36]. Plasma exchange is thought to act by removing pathogenic antibodies, humoral mediators, and complement proteins involved in the pathogenesis of GBS. It is given as a volume of exchange over five sessions. Both treatment modalities have been shown to be equally effective [37]. Although an effect is noted if either treatment is given within four weeks, a stronger effect may be elicited if treatment is administered within two weeks [38-40]. Corticosteroids have not shown a benefit over placebo or in combination with IVIG and plasma exchange. Treatment is generally considered to shorten the course of recovery from GBS [37,39,41-44].

Our patient initially suffered from a category I COVID-19 infection, which is defined as a patient who is either asymptomatic or with features of fever and symptoms of upper respiratory tract infection according to the AIIMS/ICMR National Task Force Classification [45]. Twelve days after her fever subsided, she developed demyelinating symptoms suggestive of GBS. Because she presented after two weeks of disease onset with static symptoms, the decision to administer IVIG was deferred [46] and she was put on conservative management, and she had a full recovery. Spontaneous resolution of the clinical manifestations of GBS is rare. Self-limitation of the disease course can occur in some patients owing to a spontaneous repair mechanism [47].

Because there has been a rise in patients presenting with post-COVID-19 sequelae, there should be a high suspicion of GBS among patients presenting with neurological manifestations. The literature review highlights the co-existence of the SARS-CoV-2 infection and GBS and emphasizes its early diagnosis and treatment.

## Conclusions

It is becoming more evident that COVID-19 is a multisystem disease with dysregulated immune response. This study reinforces the idea that there is an association between COVID-19 infection and GBS via an autoimmune cross-reactivity mechanism. Close attention should be paid to neurologic complications such as GBS in COVID-19 patients, and early detection of symptoms and diagnosis are important. According to the literature review, GBS following COVID-19 was more severe, urging us to include detailed neurological examination in COVID-19 patients.
